# New-onset metabolic syndrome is associated with accelerated renal function decline partially through elevated uric acid: an epidemiological cohort study

**DOI:** 10.3389/fendo.2024.1328404

**Published:** 2024-02-02

**Authors:** Qiuyu Xu, Xiaohong Fan, Gang Chen, Jie Ma, Wenling Ye, Sanxi Ai, Li Wang, Ke Zheng, Yan Qin, Limeng Chen, Mingxi Li, Xuemei Li

**Affiliations:** ^1^ Department of Nephrology, Peking Union Medical College Hospital, Chinese Academy of Medical Sciences, Beijing, China; ^2^ 4^ + ^4 Medical Doctor Program, Chinese Academy of Medical Sciences & Peking Union Medical College, Beijing, China; ^3^ Department of Epidemiology and Biostatistics, Institute of Basic Medical Sciences Chinese Academy of Medical Sciences, School of Basic Medicine Peking Union Medical College, Beijing, China

**Keywords:** metabolic syndrome, chronic kidney disease, hyperuricemia, hypertension, diabetes mellitus, obesity

## Abstract

**Background:**

The burden of metabolic syndrome (MetS) continues to rise globally and is associated with complications of multiple organ systems. We aimed to identify the association between changes in MetS status and accelerated renal function progression through a regional epidemiological survey in China, thus discovering influence factors with treatable potential.

**Methods:**

This study was a population-based survey conducted in 2008 and 2014, assessing a representative sample of 5,225 individuals from rural areas of China. They were divided into four subgroups according to their MetS status in 2008 and 2014 (Never, Previously abnormal, New-onset, and Consistent). Multivariate logistic regression and stratification analysis evaluated the relationship between clinical factors and renal function decline under different MetS statuses. Smooth curve fitting further addressed the role of serum uric acid, illustrating the vital turning point of uric acid levels in the background of renal function deterioration.

**Results:**

Of all groups of MetS states, the new-onset MetS showed the most significant eGFR decline, with a 6.66 ± 8.21 mL/min/1.73 m^2^ decrease over 6 years. The population with newly-onset MetS showed a considerable risk increase in delta eGFR with a beta coefficient of 1.66 (95%CI=1.09-2.23) after necessary correction. In searching for the drivers, the strength of the association was significantly reduced after additional adjustment for uric acid levels (β=0.91, 95%CI=0.35-1.45). Regarding the turning point, uric acid levels exceeding 426 μmol/L were more significantly associated with the stepped-up deterioration of kidney function for those with new-onset MetS.

**Conclusion:**

Metabolic syndrome demonstrated a solid correlation with the progression of renal function, particularly in those with newly-onset MetS status. In addition to the diagnostic components of MetS, hyperuricemia could be used as a marker to identify the high risk of accelerating eGFR decline early. Furthermore, we suggested a potential renal benefit for the newly-onset MetS population when maintaining their serum uric acid level below the criteria for asymptomatic hyperuricemia.

## 1 Introduction

Metabolic syndrome (MetS), with globally increasing prevalence (1), is characterized by cardiovascular risk factors, such as abdominal obesity, insulin resistance, hypertension, impaired glucose metabolism, and dyslipidemia. MetS is known to be with increased cardiovascular disease (CVD) risk in the general population (2), along with incident overt type 2 diabetes (3), non-alcoholic fatty liver disease (4), and hyperuricemia (5). The kidney is one of the established target organs damaged by metabolic factors. MetS is essential in new-onset chronic kidney disease (CKD) and CKD progression (6, 7). Although many studies associate MetS with CKD, causality remains unproven (8).

Previous observational studies have reported an independent association between MetS and proteinuria and renal function decline (9–11). However, the risk estimates for developing clinical outcomes with MetS and its components differed among these studies, some reporting statistically insignificant associations, especially when the sample was predominantly Asian (10, 12, 13). Diabetes and hypertension are the worldwide leading causes of development of both CKD and end-stage renal disease (ESRD), as well as in contemporary China (14). Since both impaired fasting glucose and elevated blood pressure (BP) are included in the definition of MetS, it would be essential to explore the impact of the other three MetS components or other metabolic factors outside the diagnostic criteria that could be intervened at an early stage in the context of slowing renal function decline.

Hyperuricemia is significantly associated with the development and severity of metabolic syndrome (15), while it is not currently included in the diagnostic criteria for MetS. Epidemiological studies showed that hyperuricemia independently predicts the development of CKD in individuals with normal kidney function (16, 17). However, not all studies have demonstrated a significant association between elevated serum uric acid levels and the incidence of CKD risk (18). The role of uric acid may be more challenging to identify in the context of multiple risk factors and pathogenetic mechanisms typical of overt CKD, such as proteinuria and high blood pressure. The discrepancy in clinical results raised whether urate-lowering treatment could provide a constant benefit in all patients with hyperuricemia and CKD (19, 20). This put forward the need to screen out particular populations more likely to benefit from intervention and determine the threshold values of serum uric acid above which intervention could be suggested.

Even though collectively, there is ample observational data to support an association between the MetS and CKD independent of hypertension or diabetes, limited attention has been directed towards understanding the impact of altered metabolic status on kidney function. Moreover, the earlier stages of CKD are typically missed because of their asymptomatic nature. To further the potential association of the MetS with CKD, we conducted an epidemiological investigation in rural China, an area known to have a high incidence of metabolic disease (21), to search for intervenable drivers behind the association between metabolic syndrome status change and decreased kidney function.

## 2 Materials and methods

### 2.1 Study population

The study population consisted of 6,925 adults recruited during two different periods from the Pinggu District, a suburban community in Beijing, PR. China. The target population was residents aged 30–75 years in 2008. Multistage clustered sampling was used to recruit study participants, with the probability proportional to population size in each stage. Details of multistage clustered sampling with the probability proportional to population size have been previously described (21, 22).

**Figure 1:**
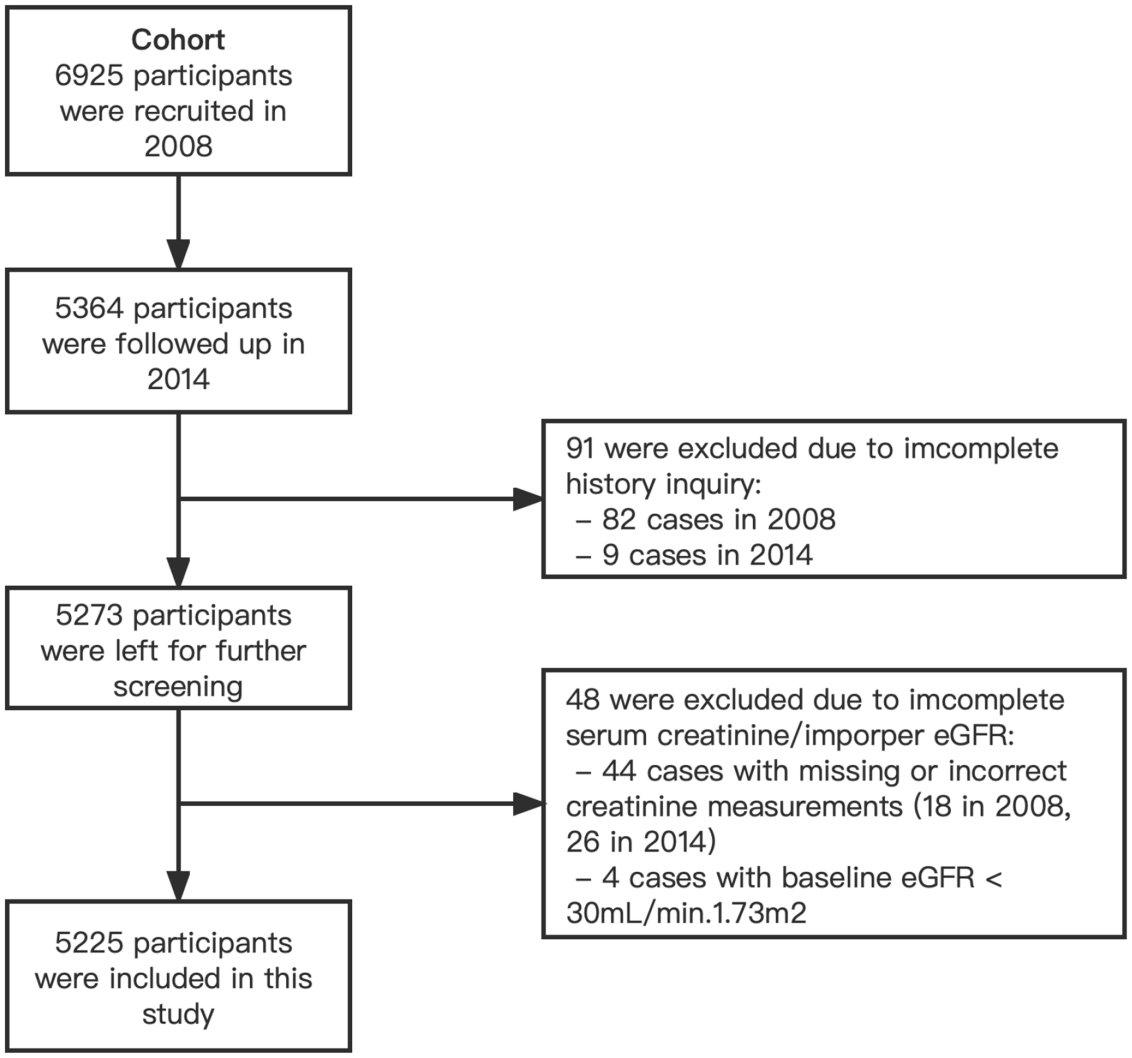
Flowchart of study population recruitment.

In the Cohort, 6,925 participants from 20 villages were enrolled and underwent a first study examination between April 2008 and March 2009. 5,364 (77.5%) of the participants returned for a second examination in 2014, and 5,273 had complete demographic data and history inquiries. 48 participants were excluded due to incomplete serum creatinine to calculate the estimated glomerular filtration rate (eGFR). Considering that the relevant indicators of participants with advanced CKD and those who had started dialysis might deviate significantly from the population average and cause significant interference to the study analysis, 4 participants with eGFR less than 30 mL/min/1.73 m^2^ in 2008 were excluded from the study. We finally included 5,225 subjects as a cohort in the current study (**Figure 1**).

The Peking Union Medical College Hospital (PUMCH) Ethics Committee approved the study. Written informed consent was obtained from each participant before data collection.

### 2.2 Definitions

#### 2.2.1 Metabolic syndrome

The definition of metabolic syndrome is based on criteria recommended in the National Cholesterol Education Program Adult Treatment Panel III (NCEP ATP III) guidelines with a modification (23, 24), which is widely used in China and other East Asian countries (25, 26). Metabolic syndrome was identified by the presence of three or more of the following components: abdominal obesity, hypertriglyceridemia (serum triglyceride level≥1.69 mmol/L), low HDL cholesterol level (HDL<1.29 mmol/L in women and <1.03 mmol/L in men), elevated blood pressure (average BP≥130/85 mmHg or on antihypertensive medications), and impaired glucose metabolism (presence of diabetes, use of oral hypoglycemics, or fasting blood glucose level ≥5.6 mmol/L). Specifically, abdominal obesity is defined as a waist circumference greater than 90 cm in men and greater than 80 cm in women, according to the International Obesity Task Force central obesity criteria for Asians (27).

#### 2.2.2 Metabolic syndrome status

The participants were divided into four subgroups according to their disease status in 2008 and 2014. The ‘Never’ group was defined as those who did not meet the diagnostic criteria for MetS in either 2008 or 2014. Participants diagnosed with MetS in 2008 but did not meet the diagnosis in 2014 after lifestyle or pharmacological interventions were classified as the ‘Previously abnormal’ group. The ‘New-onset’ group referred to the population who did not meet a MetS diagnosis in 2008 and were newly diagnosed with MetS in 2014. Finally, those with persistent MetS status in 2008 and 2014 were labeled as the ‘Consistent’ group.

#### 2.2.3 CKD and delta eGFR

GFR was estimated using the simplified prediction equation derived from the Chronic Kidney Disease Epidemiology Collaboration (CKD-EPI) (28). CKD is defined as an eGFR less than 60 mL/min/1.73 m^2^ (<1.00 mL/s/1.73 m^2^) according to the Kidney Disease: Improving Global Outcomes (KDIGO) 2012 Clinical Practice Guideline (29). The delta eGFR was calculated by estimating the difference in eGFR between 2008 and 2014 based on serum creatinine levels.

### 2.3 Measurements

All participants completed a questionnaire to document demographic information and self-reported living style status, previous medical history, family history, and medication history. Staff was trained to measure height, body weight, and circumference by a standardized method. BP was measured twice after at least 5 minutes of rest using a mercury sphygmomanometer. If the difference between the two measurements was >10 mmHg, a third measurement was obtained, and the mean value of the BPs was calculated using the two closest values.

Venous blood samples were collected after overnight fasting of 8 to 12 hours. The concentration of fasting blood glucose, serum creatinine, serum inorganic ions, uric acid, total cholesterol, low-density lipoprotein cholesterol, high-density lipoprotein cholesterol, and triglyceride were measured using a Beckman Coulter AU5800 (Brea, CA), and serum creatinine was determined and calibrated by an enzymatic method. Hemoglobin was measured using the SYSMEX-500 (Kobe, Japan). Plasma glycosylated hemoglobin (HbA1c) was detected by VARIANT II Turbo (Hercules, CA).

First void morning urine was collected to detect the albumin-to-creatinine ratio (ACR) using a Beckman Coulter AU270. Urine albumin was measured with the immunoturbidimetric method, and urine creatinine (UCr) was measured with an enzymatic method. Calibrated UCr was used for ACR; 200 urine samples were retested by Jaffe’s kinetic method, and the following calibration equation was approved (R2 = 0.997): calibrated UCr (mmol/l) = 0.884 UCr (mmol/l) + 0.3355.

All blood and urine samples were transferred at 4°C in iceboxes and tested at the PUMCH laboratory.

### 2.4 Statistical analyses

According to metabolic syndrome status, demographic data and laboratory measurements from two cross-sections were divided into four groups (Never, Previously abnormal, New-onset, and Consistent). The summarized statistical results were expressed as counts and percentages for the categorical data, and means with standard deviation were determined for continuous variables with approximately normal distributions. Differences between the four groups were compared using chi-square and one-way analysis of variance (ANOVA) for categorical and continuous variables, respectively. We performed univariate analysis to determine the effect of related factors on the prognosis of renal function. The criteria for variable inclusion in the following regression analysis were based on both statistical significance in univariate analysis and clinical relevance. To ensure the clinical relevance of the selected variables, we conducted a comprehensive evaluation through collaborative discussions involving experienced clinicians and statistical experts. We further remove the variables with strong multicollinearity in the multiple regression analysis.

Multivariate logistic regression and stratification analysis were conducted to investigate the relationship between clinical factors and renal function decline under different MetS statuses. Subgroup analysis was performed by stratified multiple regression analysis. Finally, we used smooth curve fitting and generalized additive models to address the nonlinear relationship between serum uric acid and renal function decline in different MetS status subgroups. For non-linearity in the model, a recursive algorithm was used to calculate the inflection point when non-linearity was detected, with a two-segmented linear regression model on either side of the inflection point. All data analyses and graphs were performed using R 4.2.0 (http://www.r-project.org) and EmpowerStats 4.1 epidemiology program (www.empowerstats.com).

## 3 Results

A total of 5,225 participants were included in our analysis. The weighted characteristics of the participants were subdivided according to their MetS status in 2008 and 2014, and their baseline characteristics are described in **Tables 1**, **2**. Compared with other participants, the Consistent group was more likely to be female, self-reporting more cardiovascular diseases, and having a family history of obesity, hypertension, and diabetes (**Table 1**). Notably, the number of participants reporting prior gout in the overall population was quite limited. As for the laboratory tests, the Consistent group was more inclined to have higher uric acid levels, fasting glucose, hemoglobin A1c, total cholesterol (TC), triglyceride (TG), and urine ACR. It was worth mentioning that the New-onset group had the highest eGFR decline from 2008 to 2014, even exceeding the Consistent group (**Table 2**). The distribution of the delta eGFR in each subgroup was presented as a bar chart and violin plot in **Supplementary Figure 1**.

**Table 1:** Geographic features of the follow-up population in 2014 after the 2008 epidemiology study.

Metabolic syndrome status	Never (n=2545)	Previously abnormal (n=491)	New-onset (n=821)	Consistent (n=1354)	P-value
Age at 2014	55.00 ± 10.01	55.09 ± 9.41	54.97 ± 10.03	56.49 ± 9.44	<0.001
Male	1428 (56.11)	212 (43.18)	368 (44.82)	473 (34.93)	<0.001
Self-reported disease histories					
Kidney stone in 2008	182 (7.15)	34 (6.92)	55 (6.70)	123 (9.08)	0.099
Kidney stone in 2014	211 (8.31)	49 (10.02)	80 (9.74)	143 (10.56)	0.110
Gout in 2008	5 (0.20)	0 (0.00)	3 (0.37)	4 (0.30)	0.540
Gout in 2014	18 (0.71)	4 (0.82)	5 (0.61)	26 (1.92)	0.002
Cardiovascular diseases 2008	131 (5.15)	51 (10.39)	69 (8.40)	138 (10.19)	<0.001
Cardiovascular diseases 2014	159 (6.26)	52 (10.63)	76 (9.26)	173 (12.78)	<0.001
Cerebrovascular diseases 2008	93 (3.65)	24 (4.89)	34 (4.14)	86 (6.35)	0.002
Cerebrovascular diseases 2014	152 (5.99)	45 (9.20)	63 (7.67)	120 (8.86)	0.003
Family histories					
Obese	234 (9.19)	82 (16.50)	100 (12.18)	218 (16.10)	<0.001
Hypertension	908 (35.68)	230 (46.84)	344 (41.90)	679 (50.15)	<0.001
Diabetes	248 (9.74)	66 (13.44)	112 (13.64)	219 (16.17)	<0.001
Hyperlipidemia	1 (0.04)	0 (0.00)	3 (0.37)	0 (0.00)	0.013
Gout	1 (0.04)	0 (0.00)	1 (0.12)	0 (0.00)	0.532
Cardiovascular diseases	523 (20.55)	113 (23.01)	181 (22.05)	307 (22.67)	0.364
Cerebrovascular diseases	841 (33.05)	193 (39.31)	312 (38.00)	541 (39.96)	<0.001
Chronic kidney diseases	63 (2.48)	16 (3.26)	28 (3.41)	42 (3.10)	0.421
Cancer	371 (14.58)	74 (15.07)	119 (14.49)	204 (15.07)	0.969

**Table 2:** Measurements and findings in follow-up population in 2008 and 2014 epidemiology studies.

Metabolic syndrome status	Never (n=2545)	Previously abnormal (n=491)	New-onset (n=821)	Consistent (n=1354)	P-value
Body measurements					
BMI 2008	23.45 ± 2.85	26.67 ± 3.18	25.82 ± 3.05	27.78 ± 3.19	<0.001
BMI 2014	24.32 ± 3.16	26.73 ± 3.66	27.27 ± 3.06	28.25 ± 3.24	<0.001
Waist 2008	80.65 ± 8.30	90.66 ± 8.17	87.97 ± 8.34	93.77 ± 8.19	<0.001
Waist 2014	81.95 ± 8.38	88.33 ± 8.77	90.86 ± 7.46	93.20 ± 8.09	<0.001
Hip 2008	92.27 ± 5.83	97.45 ± 6.12	96.24 ± 5.83	98.81 ± 6.00	<0.001
Hip 2014	92.65 ± 6.02	96.21 ± 6.64	97.92 ± 5.97	99.09 ± 6.35	<0.001
Mean MAP 2008	98.61 ± 11.96	105.68 ± 11.54	100.80 ± 11.31	107.76 ± 11.28	<0.001
Mean MAP 2014	103.87 ± 12.88	108.36 ± 13.83	111.18 ± 11.60	113.99 ± 12.48	<0.001
Laboratory tests					
WBC 2014	6.34 ± 1.68	6.65 ± 1.93	6.56 ± 1.64	6.94 ± 1.82	<0.001
Hemoglobin 2014	148.14 ± 15.36	148.03 ± 16.13	148.74 ± 15.57	147.65 ± 15.36	0.466
Platelet 2014	248.13 ± 64.17	252.15 ± 61.06	252.78 ± 72.89	255.16 ± 66.24	0.012
ALT (U/L) 2008	19.00 ± 15.06	23.63 ± 15.67	21.19 ± 15.55	23.66 ± 15.05	<0.001
ALT (U/L) 2014	17.78 ± 11.35	19.76 ± 14.17	21.40 ± 14.54	21.56 ± 13.41	<0.001
Albumin (g/L) 2008	46.75 ± 2.57	47.11 ± 2.58	46.69 ± 2.68	46.91 ± 2.59	0.010
Albumin (g/L) 2014	44.45 ± 2.40	44.96 ± 2.44	44.55 ± 2.54	44.83 ± 2.43	<0.001
Serum creatinine 2008	63.72 ± 12.89	63.21 ± 13.30	64.58 ± 15.28	64.01 ± 15.29	0.304
Serum creatinine 2014	65.58 ± 12.85	63.90 ± 12.64	69.24 ± 33.03	67.52 ± 48.29	0.002
Urea 2008	5.25 ± 1.47	4.87 ± 1.28	5.55 ± 1.52	5.23 ± 1.43	<0.001
Urea 2014	5.34 ± 1.48	4.88 ± 1.22	5.90 ± 1.84	5.56 ± 1.90	<0.001
Uric acid 2008	233.93 ± 63.82	256.91 ± 75.37	250.11 ± 71.65	268.04 ± 75.28	<0.001
Uric acid 2014	256.91 ± 70.77	271.85 ± 80.31	286.07 ± 82.49	292.04 ± 83.13	<0.001
Fasting glucose 2008	5.41 ± 1.06	6.17 ± 1.77	5.44 ± 1.35	6.26 ± 1.98	<0.001
Fasting glucose 2014	5.90 ± 1.26	6.69 ± 2.26	6.04 ± 1.40	7.04 ± 2.36	<0.001
Total cholesterol 2008	4.86 ± 0.89	5.16 ± 1.14	5.03 ± 0.93	5.24 ± 1.02	<0.001
Total cholesterol 2014	4.74 ± 0.84	4.89 ± 0.92	4.92 ± 0.91	5.00 ± 0.99	<0.001
Triglyceride 2008	1.03 ± 0.71	1.97 ± 1.94	1.25 ± 0.86	2.46 ± 1.97	<0.001
Triglyceride 2014	0.93 ± 0.54	1.17 ± 0.64	1.64 ± 1.26	2.25 ± 1.88	<0.001
HDL 2008	1.48 ± 0.33	1.23 ± 0.28	1.34 ± 0.28	1.13 ± 0.22	<0.001
HDL 2014	1.48 ± 0.32	1.35 ± 0.26	1.24 ± 0.29	1.13 ± 0.22	<0.001
LDL 2008	2.93 ± 0.77	3.20 ± 0.87	3.19 ± 0.79	3.23 ± 0.85	<0.001
LDL 2014	2.90 ± 0.75	3.13 ± 0.79	3.09 ± 0.77	3.09 ± 0.83	<0.001
HbA1C 2014	5.63 ± 0.72	6.14 ± 1.26	5.79 ± 0.80	6.34 ± 1.24	<0.001
Urine ACR (mg/g) 2008	13.71 ± 33.33	16.25 ± 23.08	19.14 ± 64.79	22.97 ± 65.95	<0.001
Urine ACR (mg/g) 2014	17.25 ± 102.47	18.70 ± 48.14	36.44 ± 289.18	41.60 ± 217.45	<0.001
eGFR (mL/min/1.73m²) 2008	104.76 ± 11.79	103.39 ± 11.97	102.60 ± 13.14	100.61 ± 13.76	<0.001
eGFR (mL/min/1.73m²) 2014	99.40 ± 11.36	99.10 ± 11.57	95.94 ± 14.85	95.30 ± 15.01	<0.001
Delta eGFR (2008 vs. 2014)	5.36 ± 7.07	4.29 ± 7.27	6.66 ± 8.21	5.31 ± 8.48	<0.001
Diagnosis and findings					
Abdominal obesity 2008	689 (27.07)	418 (85.13)	508 (61.88)	1268 (93.72)	<0.001
Abdominal obesity 2014	778 (30.81)	298 (61.57)	715 (87.41)	1256 (93.31)	<0.001
Increased TG 2008	182 (7.15)	266 (54.18)	99 (12.06)	883 (65.21)	<0.001
Increased TG 2014	120 (4.72)	32 (6.52)	323 (39.34)	753 (55.61)	<0.001
Decreased HDL 2008	304 (11.94)	254 (51.73)	175 (21.32)	934 (68.98)	<0.001
Decreased HDL 2014	267 (10.49)	88 (17.92)	419 (51.04)	952 (70.31)	<0.001
Elevated blood pressure 2008	866 (34.03)	357 (72.71)	316 (38.49)	1081 (79.84)	<0.001
Elevated blood pressure 2014	1340 (52.65)	318 (64.77)	730 (88.92)	1244 (91.88)	<0.001
Abnormal glucose 2008	695 (27.31)	342 (69.65)	175 (21.32)	830 (61.30)	<0.001
Abnormal glucose 2014	925 (36.35)	107 (21.79)	542 (66.02)	755 (55.76)	<0.001
New-onset CKD in 2008	168 (6.60)	56 (11.41)	80 (9.74)	215 (15.88)	<0.001
New-onset CKD in 2014	188 (7.39)	52 (10.59)	93 (11.33)	230 (16.99)	<0.001
New-onset abdominal obesity	236 (9.35)	4 (0.83)	214 (26.16)	45 (3.34)	<0.001
New-onset increased TG	80 (3.14)	4 (0.81)	250 (30.45)	144 (10.64)	<0.001
New-onset abnormal BP	623 (24.48)	37 (7.54)	419 (51.04)	200 (14.77)	<0.001
New-onset abnormal BG	597 (23.46)	17 (3.46)	409 (49.82)	250 (18.46)	<0.001
New-onset decreased HDL	125 (4.87)	7 (1.43)	267 (32.52)	141 (10.41)	<0.001

BMI, Body Mass Index; MAP, Mean Artery Pressure; WBC, White Blood Cell; ALT, Alanine Aminotransferase; HDL, High-Density Lipoprotein; LDL, Low-Density Lipoprotein; HbA1C, Hemoglobin A1c; ACR, Albumin-to-Creatinine Ratio; eGFR, estimated Glomerular Filtration Rate; TG, Triglyceride; CKD, Chronic Kidney Disease; BP, Blood Pressure; BG, Blood Glucose.

We performed a univariate analysis of renal function decline (**Supplementary Table 1**). Compared with the Never group, the New-onset group showed a significant increase of risk in delta eGFR with a beta coefficient of 1.30 (95%CI: 0.70, 1.90), while the beta coefficient of the other two groups was -1.07 (95%CI: -1.81, -0.33) for the Previously abnormal group, and -0.05 (95%CI: -0.55, 0.46) for the Consistent group. Further multivariable regression analysis provided a better understanding of the relationship between metabolic syndrome status and renal function decline, along with its underlying driver (**Table 3**). The association between New-onset MetS in 2014 and delta eGFR was significant both in the non-adjusted model (β=1.30, 95%CI=0.70-1.90, P<0.001) and the model adjusted for gender and age (Adjust I: β=1.22, 95%CI=0.61-1.82, P<0.001). The model remained significant when additional adjusted covariates such as blood pressure, BMI, Urine ACR, albumin, glucose, TG, etc., were included (Adjust II: β=1.66, 95%CI=1.09-2.23, P<0.001). Still, the strength of the association was significantly reduced after additional adjustment for uric acid levels (Adjust III: β=0.91, 95%CI=0.37-1.45, P=0.001), suggesting that uric acid is a vital mediating factor between new-onset MetS status and decreased renal function. We also stratified by continuous and categorical variables (terciles) (**Table 4**). Conclusions and variation trends remained consistent in the stratified analysis of each factor.

**Table 3:** Logistic regression for delta eGFR and metabolic syndrome status.

Exposure for delta eGFR	Non-adjusted β (95%CI), P	Adjust I β (95%CI), P	Adjust II β (95%CI), P	Adjust III β (95%CI), P
**Metabolic syndrome status**	N=5211	N=5211	N=4968	N=4968
** Never**	Reference	Reference	Reference	Reference
** Previously abnormal**	-1.07 (-1.81, -0.33), 0.005	-1.17 (-1.91, -0.43), 0.002	-0.34 (-1.07, 0.39) 0.366	-0.33 (-1.02, 0.36) 0.346
** New-onset**	1.30 (0.70, 1.90), <0.001	1.22 (0.61, 1.82), <0.001	1.66 (1.09, 2.23) <0.001	0.91 (0.37, 1.45) 0.001
** Consistent**	-0.05 (-0.55, 0.46), 0.852	-0.27 (-0.78, 0.25), 0.306	0.82 (0.21, 1.43) 0.008	0.23 (-0.35, 0.80) 0.435

Adjust I model adjusted for: Gender, Age in 2014.

Adjust II model adjusted for: Gender, Age in 2014, Mean MAP 2008, BMI 2008, Urine ACR (mg/g) enzyme 2008, Albumin (g/L) 2008, SCr enzyme 2008, Glucose 2008, LDL 2008, TG 2008, Uric acid 2008, Kidney stone 2008, Albumin 2014, Glucose 2014, Uric acid 2014, Urine ACR (mg/g) 2014, Kidney stone 2014.

Adjust III model adjusted for: All factors in adjust II except Uric acid 2014.

eGFR, estimated Glomerular Filtration Rate; MAP, Mean Artery Pressure; BMI, Body Mass Index; ACR, Albumin-to-Creatinine Ratio; SCr, Serum Creatinine; LDL, Low-Density Lipoprotein; TG, Triglyceride.

**Table 4:** Stratification analysis for delta eGFR in different metabolic syndrome status groups.

Y= Delta eGFR	N	Never	Previously abnormal	New-onset	Consistent
Gender					
Female	2730	0	-0.99 (-1.97, 0.00) 0.0502	0.86 (0.04, 1.68) 0.0400	-0.03 (-0.69, 0.63) 0.9291
Male	2481	0	-1.38 (-2.50, -0.25) 0.0165	1.67 (0.77, 2.56) 0.0003	-0.50 (-1.31, 0.31) 0.2279
Age					
35 - 49	1511	0	-1.26 (-2.54, 0.01) 0.0525	0.60 (-0.39, 1.60) 0.2355	-0.84 (-1.75, 0.06) 0.0688
50 - 58	1812	0	-0.85 (-2.01, 0.32) 0.1536	1.33 (0.37, 2.30) 0.0067	-0.45 (-1.25, 0.35) 0.2697
59 - 81	1888	0	-1.14 (-2.51, 0.23) 0.1037	1.91 (0.77, 3.04) 0.0011	0.79 (-0.12, 1.70) 0.0901
Mean MAP in 2008					
60.8 - 96	1711	0	-1.49 (-3.01, 0.04) 0.0568	0.52 (-0.40, 1.44) 0.2692	-1.95 (-3.06, -0.85) 0.0006
96.3 - 106.3	1719	0	-0.51 (-1.70, 0.69) 0.4054	1.21 (0.27, 2.15) 0.0121	-0.00 (-0.86, 0.85) 0.9983
106.7 - 181.7	1772	0	-1.83 (-3.12, -0.54) 0.0054	2.41 (1.10, 3.72) 0.0003	-0.24 (-1.15, 0.67) 0.6099
BMI in 2008					
15.27 - 23.49	1736	0	-0.73 (-2.60, 1.14) 0.4430	1.56 (0.37, 2.75) 0.0103	1.99 (0.44, 3.55) 0.0122
23.49 - 26.56	1734	0	-0.35 (-1.56, 0.86) 0.5708	1.74 (0.77, 2.72) 0.0005	-0.53 (-1.45, 0.40) 0.2638
26.56 - 44.61	1740	0	-1.08 (-2.36, 0.20) 0.0977	1.32 (0.14, 2.51) 0.0290	0.55 (-0.42, 1.52) 0.2689
Uric acid in 2008					
78 - 210	1683	0	-0.89 (-2.09, 0.31) 0.1447	0.82 (-0.06, 1.70) 0.0695	-0.44 (-1.28, 0.40) 0.3080
211 - 269	1688	0	-0.63 (-1.88, 0.63) 0.3277	1.38 (0.34, 2.43) 0.0097	0.49 (-0.37, 1.35) 0.2609
270 - 615	1692	0	-0.63 (-2.03, 0.77) 0.3777	2.17 (0.95, 3.38) 0.0005	0.82 (-0.15, 1.80) 0.0989
Uric acid in 2014					
73 - 231	1719	0	-1.28 (-2.25, -0.30) 0.0102	0.12 (-0.72, 0.96) 0.7795	-0.86 (-1.59, -0.14) 0.0200
232 - 293	1730	0	-1.37 (-2.58, -0.17) 0.0257	1.04 (0.02, 2.05) 0.0458	-0.55 (-1.40, 0.31) 0.2093
294 - 786	1762	0	-0.73 (-2.33, 0.86) 0.3680	1.91 (0.71, 3.12) 0.0019	0.28 (-0.72, 1.29) 0.5803
Serum creatinine in 2008					
7 - 55	1583	0	-0.79 (-1.89, 0.31) 0.1595	-0.01 (-0.94, 0.91) 0.9774	-0.25 (-1.00, 0.50) 0.5083
56 - 68	1837	0	-1.39 (-2.46, -0.31) 0.0114	1.15 (0.29, 2.02) 0.0092	-0.00 (-0.74, 0.74) 0.9958
69 – 215	1791	0	-1.23 (-2.74, 0.28) 0.1104	2.61 (1.41, 3.81) <0.0001	-0.14 (-1.16, 0.88) 0.7815
Serum creatinine in 2014					
15 - 58	1722	0	-0.98 (-1.83, -0.14) 0.0226	-0.16 (-0.90, 0.57) 0.6610	-0.82 (-1.40, -0.24) 0.0059
59 - 69	1643	0	-0.96 (-2.09, 0.17) 0.0975	0.64 (-0.33, 1.62) 0.1966	-0.45 (-1.26, 0.36) 0.2763
70 - 1499	1846	0	-0.92 (-2.62, 0.77) 0.2866	2.69 (1.47, 3.91) <0.0001	1.24 (0.16, 2.32) 0.0251
Albumin in 2008					
26 - 45	1488	0	-0.33 (-1.84, 1.17) 0.6653	1.80 (0.67, 2.93) 0.0018	0.44 (-0.52, 1.49) 0.3711
46 - 47	1687	0	-1.43 (-2.69, -0.17) 0.0265	1.29 (0.28, 2.30) 0.0124	0.23 (-0.64, 1.10) 0.6036
48 - 56	2036	0	-0.91 (-2.04, 0.22) 0.1148	0.98 (-0.01, 1.96) 0.0524	-0.43 (-1.23, 0.37) 0.2955
Albumin in 2014					
26 - 43	1596	0	-0.35 (-1.91, 1.21) 0.6606	1.87 (0.75, 2.98) 0.0010	0.41 (-0.59, 1.40) 0.4246
44 - 45	1765	0	-0.96 (-2.12, 0.20) 0.1044	0.76 (-0.28, 1.80) 0.1521	0.29 (-0.55, 1.13) 0.4966
46 - 55	1850	0	-1.49 (-2.68, -0.29) 0.0146	1.21 (0.22, 2.19) 0.0164	-0.56 (-1.37, 0.26) 0.1790
Glucose in 2008					
2.3 - 5.1	1728	0	0.11 (-1.64, 1.87) 0.9009	1.54 (0.56, 2.51) 0.0021	0.95 (-0.15, 2.04) 0.0908
5.2 - 5.6	1721	0	-0.37 (-1.76, 1.02) 0.6040	1.42 (0.52, 2.31) 0.0021	0.69 (-0.16, 1.54) 0.1133
5.7 - 22.2	1762	0	-1.28 (-2.37, -0.19) 0.0212	0.32 (-1.06, 1.69) 0.6507	-0.37 (-1.21, 0.48) 0.3941
Glucose in 2014					
3.9 - 5.5	1688	0	-0.63 (-2.00, 0.74) 0.3686	1.19 (0.25, 2.14) 0.0130	0.28 (-0.73, 1.29) 0.5819
5.6 - 6.1	1776	0	-0.63 (-1.83, 0.56) 0.3007	1.86 (0.96, 2.76) <0.0001	0.96 (0.12, 1.80) 0.0260
6.2 - 26.8	1747	0	-1.61 (-2.95, -0.28) 0.0181	0.65 (-0.69, 2.00) 0.3431	-0.67 (-1.60, 0.26) 0.1588
TC in 2008					
2.2 - 4.56	1725	0	0.23 (-1.17, 1.63) 0.7455	1.45 (0.35, 2.54) 0.0099	0.05 (-0.90, 1.00) 0.9139
4.57 - 5.36	1747	0	-1.37 (-2.63, -0.11) 0.0328	1.62 (0.66, 2.59) 0.0010	-0.49 (-1.38, 0.39) 0.2718
5.37 - 12.96	1739	0	-1.48 (-2.70, -0.26) 0.0176	1.02 (-0.06, 2.10) 0.0638	0.47 (-0.38, 1.31) 0.2764
TG in 2008					
0.25 - 0.9	1731	0	0.21 (-1.35, 1.78) 0.7901	1.05 (0.11, 1.98) 0.0283	0.80 (-0.55, 2.14) 0.2468
0.91 - 1.5	1729	0	-1.05 (-2.58, 0.48) 0.1773	1.30 (0.36, 2.24) 0.0070	0.16 (-0.89, 1.22) 0.7603
1.51 - 34.02	1751	0	-0.33 (-1.60, 0.93) 0.6053	2.28 (0.81, 3.75) 0.0024	0.92 (-0.09, 1.94) 0.0749
LDL in 2008					
0.26 - 2.69	1723	0	0.66 (-0.65, 1.98) 0.3238	1.93 (0.85, 3.01) 0.0005	-0.16 (-1.03, 0.71) 0.7191
2.7 - 3.36	1749	0	-2.08 (-3.38, -0.77) 0.0018	1.25 (0.22, 2.28) 0.0179	-0.19 (-1.12, 0.73) 0.6848
3.37 - 8.07	1739	0	-1.08 (-2.34, 0.17) 0.0899	1.18 (0.12, 2.23) 0.0288	0.45 (-0.42, 1.32) 0.3150
Urine ACR in 2008					
0.16 - 7.6	1733	0	-0.62 (-1.84, 0.60) 0.3191	1.19 (0.21, 2.17) 0.0171	-1.05 (-1.96, -0.13) 0.0252
7.63 - 12.18	1727	0	-1.24 (-2.36, -0.12) 0.0309	0.94 (0.08, 1.80) 0.0320	-1.06 (-1.81, -0.31) 0.0054
12.19 - 1884.95	1733	0	-1.67 (-3.13, -0.21) 0.0250	1.42 (0.14, 2.70) 0.0293	0.46 (-0.53, 1.45) 0.3590
Urine ACR in 2014					
0 - 6.1	1697	0	-0.98 (-2.24, 0.28) 0.1277	1.16 (0.14, 2.18) 0.0261	-0.66 (-1.63, 0.31) 0.1836
6.19 - 10.78	1725	0	-0.73 (-1.83, 0.37) 0.1926	1.08 (0.17, 2.00) 0.0201	-0.24 (-1.00, 0.51) 0.5279
10.87 - 4743.19	1716	0	-1.26 (-2.71, 0.19) 0.0890	1.41 (0.23, 2.58) 0.0190	0.52 (-0.41, 1.46) 0.2728
Urine ACR fluctuation					
-1867 - -3.01	1708	0	-1.51 (-2.72, -0.30) 0.0147	1.14 (0.08, 2.20) 0.0359	-0.20 (-1.07, 0.66) 0.6431
-3 - 0.89	1705	0	-0.43 (-1.45, 0.95) 0.6838	0.97 (0.07, 1.87) 0.0356	-0.72 (-1.54, 0.10) 0.0848
0.9 - 4553.83	1707	0	-1.41 (-2.82, 0.00) 0.0510	1.40 (0.26, 2.54) 0.0163	0.32 (-0.60, 1.24) 0.4992

eGFR, estimated Glomerular Filtration Rate; MAP, Mean Artery Pressure; BMI, Body Mass Index; TC, Total Cholesterol; TG, Triglyceride; LDL, Low-Density Lipoprotein; ACR, Albumin-to-Creatinine Ratio.

**Figure 2:**
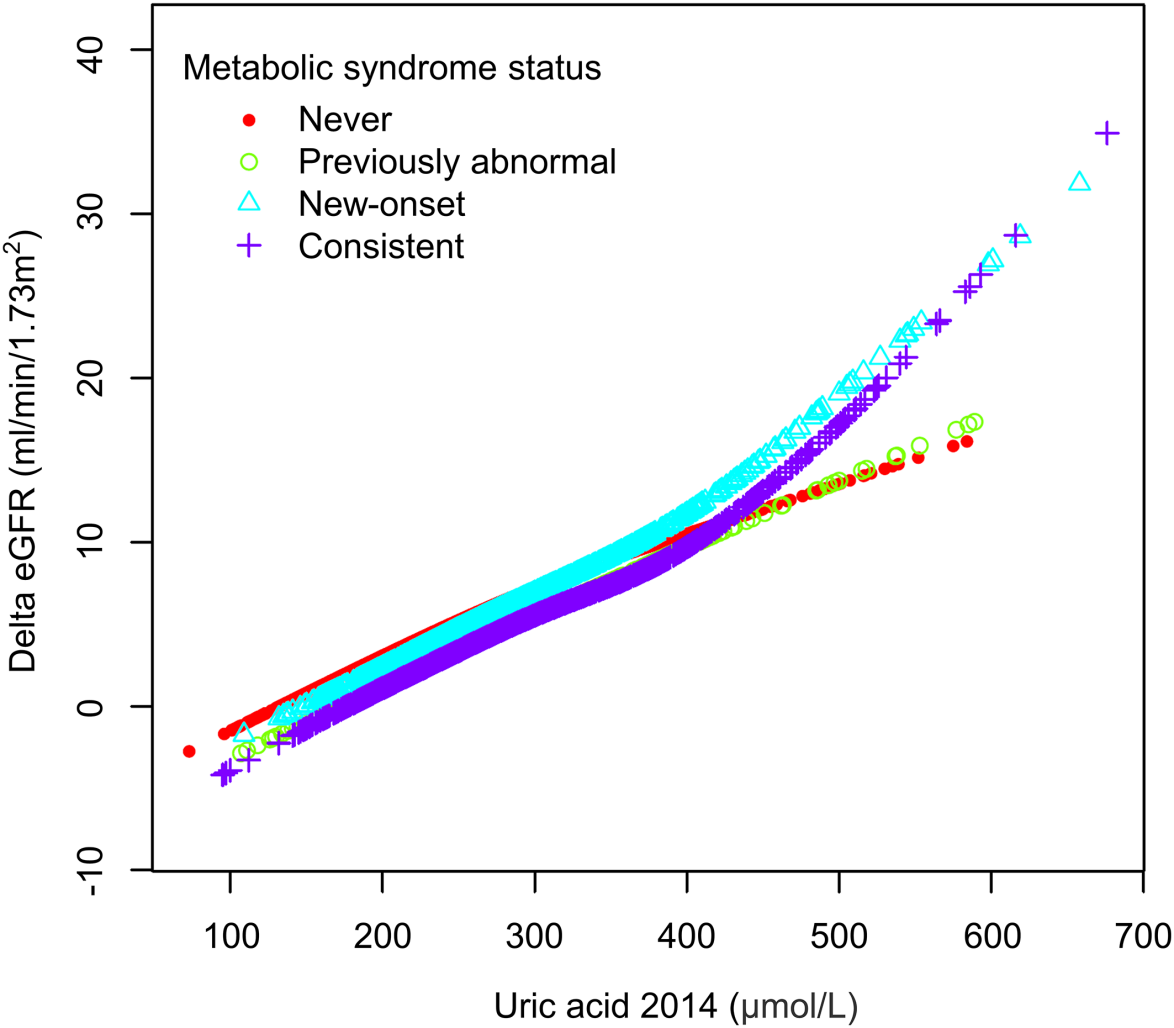
The association between uric acid and delta eGFR in different metabolic syndrome status subgroups.

We then performed weighted generalized additive models and smoothing curve fitting to assess the role of uric acid levels in the decline of renal function under different MetS statuses. Under smooth curve fitting, uric acid levels were approximately positively correlated with renal function decline, but the slopes were slightly different at lower and higher uric acid levels (**Supplementary Figure 2**). In different MetS status groups, the two-segmented relationship was most evident in the New-onset and Consistent groups. When the uric acid was relatively high, the renal function decline in the New-onset MetS population was most remarkable with the uric acid level increase (**Figure 2**). To discover the turning points, we conducted the threshold effect analysis (**Table 5**). The log-likelihood ratio test comparing the one-line (non-segmented) model to the segmented regression model was used to determine whether a threshold existed. Except for the Previously abnormal group, the goodness of fit of the two-segmented model in the other three groups was better than that of the linear model. For the population with New-onset MetS, the turning point of uric acid was 426 μmol/L, indicating that when the level of uric acid exceeded this threshold, it was more significantly associated with the decline of kidney function.

**Table 5:** Threshold effect analysis of uric acid on delta eGFR.

Metabolic syndrome status	Never	Previously abnormal	New-onset	Consistent	Total
Model I					P-interaction: <0.001
One line^*^	0.38 (0.33, 0.43) <0.0001	0.39 (0.29, 0.48) <0.0001	0.51 (0.43, 0.59) <0.0001	0.45 (0.38, 0.52) <0.0001	0.44 (0.40, 0.47) <0.0001
Model II					P-interaction: <0.001
K	332	214	426	373	413
< K 1^*^	0.44 (0.38, 0.50) <0.0001	0.60 (0.27, 0.94) 0.0005	0.46 (0.36, 0.55) <0.0001	0.39 (0.30, 0.48) <0.0001	0.41 (0.37, 0.45) <0.0001
> K 2^*^	0.24 (0.14, 0.34) <0.0001	0.36 (0.26, 0.46) <0.0001	0.81 (0.55, 1.07) <0.0001	0.56 (0.43, 0.69) <0.0001	0.58 (0.48, 0.68) <0.0001
Predict value	6.05 (5.49, 6.61)	3.75 (2.72, 4.78)	8.03 (6.79, 9.27)	5.75 (4.84, 6.65)	6.60 (6.09, 7.11)
Log-likelihood ratio test	0.003	0.180	0.014	0.037	0.003
95%CI of K	(302, 379)	NA	(371, 433)	(338, 433)	(355, 420)

When the log-likelihood ratio test P < 0.05, the two-segment model was chosen instead of the linear model.

^*^The beta coefficient corresponded to per 10 units change of serum uric acid.

## 4 Discussion

In our study, participants with new-onset MetS had a more significant decline in renal function during aging than other populations. In contrast, the eGFR decline during aging returned to the healthy population’s average level when the MetS status was corrected. By further regression analysis, we found a dose-response relation between serum uric acid and risk of eGFR loss in a Chinese rural population, and plasma uric acid level was a strong independent predictor of renal function decline in the new-onset MetS group. Smooth curve fitting and threshold analysis showed that uric acid levels above around 420 μmol/L (7 mg/dL) are associated with more accelerated eGFR decline in people with MetS.

Although the results of previous studies suggest a close association between MetS and renal function decline, it was difficult to draw a causal association and discriminate which components of MetS give rise to renal functional deterioration. Furthermore, because of the lack of evidence from clinical trials specifically involving patients with MetS, it was still unclear whether therapeutic interventions that correct one or more of the syndrome’s many features may effectively prevent harming the target organ (30). Moreover, multiple cross-sectional or cohort studies focusing on the changes in metabolic syndrome status and their impact on renal function are notably scarce. In a Korean population study, researchers evaluated the 10-year risk of CKD by examining changes in MetS status over a two-year period (31). However, limitations include categorizing participants as never, intermittent, or persistent MetS without distinguishing MetS resolution from new-onset cases in the intermittent group. Another study indicated a reduced CKD risk in those recovering from MetS, yet it defined outcome events as CKD occurrence, offering baseline creatinine levels but lacking eGFR changes (32). Therefore, from a renal protection perspective, the most common preventive strategy was identifying those at higher risk of developing CKD in this population and close monitoring to ensure the early recognition and treatment of subsequent renal abnormalities and their related complications. Our study helped identify people at a higher risk of developing CKD. In addition to the current five constituent criteria of MetS, hyperuricemia was also an essential factor associated with decreased renal function.

The biological plausibility of uric acid as a factor that can cause renal damage was supported by several *in vitro* and *in vivo* studies. Experimental studies showed that uric acid was a potent activator of the renin–angiotensin–aldosterone system, contributing to hypertension development (33). As for the cellular effect, most studies suggested that uric acid’s primary metabolic and renal effects were mediated by intracellular urate (34, 35), which was distinctly different from the processes involved in gout (36). Previous studies on the direct renal injury of uric acid mainly focused on renal tubular interstitial damage and nephrolithiasis (37). In our population, severe hyperuricemia was not very common; the number of people with gout or kidney stones was also minimal. At least from the study participants, we had a high probability of excluding the influence of their direct damage to the kidney through gout or uric acid crystals. However, the role of uric acid in the metabolic syndrome population still needs a better definition. It remains unclear whether uric acid is simply a marker, a bystander, or a key pathological element of metabolic dysregulation (38). As suggested by an Italian study, in the context of greater global risk, as is the case when renal function is even slightly reduced, serum uric acid becomes a significant correlate of cardiovascular and all-cause mortality only at serum concentration more significant than what is observed in subjects with normal renal function (39). Therefore, hyperuricemia, particularly important in the MetS population, deserves our attention.

Our study showed for the first time that the serum uric acid value had a threshold effect in people with MetS, and the turning point of the accelerated decline of renal function was consistent with the current suggested diagnostic criteria of hyperuricemia (34). The threshold was initially based on the pathogenesis of gouty arthritis, using the level beyond 6.8–7.0 mg/dL at which extracellular uric acid supersaturates to define hyperuricemia (40). Subsequent epidemiological studies employed close but slightly different cut-off values to describe the gender-specific risk of gout development, defined as above 7.7 mg/dl in men and above 6.6 mg/dl in women (41) or above 7.0 mg/dl in men and above 5.7 (42) or 6 mg/dl in women (43). Although the definition of hyperuricemia varied, meta-analysis and systematic review found that uric acid was a significant independent risk factor for the development of CKD (44). As yet, whether to treat hyperuricemia uncomplicated by articular gout, urolithiasis, or uric acid nephropathy lacks universal agreement (45). Though available epidemiological data supported the view that hyperuricemia harmed kidney function, the benefits of treatment for hyperuricemia without gout or nephrolithiasis remained controversial (46). The results’ inconsistency was partially attributed to inadequate sample size, short follow-up times, and heterogeneity in study design characterizing the randomized controlled trials.

The prevalence of metabolism-related diseases in our studied population surpasses that in the broader Chinese population (47). Several factors may contribute to this observation. Firstly, our study’s inclusion criteria targeted individuals aged 30 and above, with an average age of around 55. As the incidence of metabolism-related diseases tends to escalate with age (1), the higher average age in our sample naturally results in a greater prevalence compared to studies based on the entire age spectrum or those starting from 18 (26). Moreover, our sampled population resides in the peri-urban rural areas surrounding a rapidly developing central city in northern China. Over the past two decades, the region’s accelerated urbanization and improved economic conditions have precipitated lifestyle changes. Nevertheless, many residents lack sufficient health awareness, contributing to the adoption of unhealthy lifestyle habits (48). Considering regional factors and our population’s age distribution, our data generally aligns with previous epidemiological surveys (26, 42).

The present research had potential limitations. Firstly, as with any observational study, associations did not imply causality. However, this study would hopefully guide future interventions targeting uric acid in people with MetS to find the basis of randomized controlled trials. Besides, our study had only two cross-sections and could not continuously observe the relationship between elevated uric acid and worsening renal function. Thus, we planned to conduct a third epidemiological survey of the same scale for this population in the coming years. Thirdly, confounding variables were likely to be present. Detailed medications for treating metabolic syndrome or related conditions were not collected in our study. Besides, hyperuricemia might also reflect an unhealthy lifestyle, such as diet, drinking, and exercise. These risk factors had been confirmed to play a pathogenic role in diabetes, hypertension, and metabolic syndrome, while they were hard to quantify by epidemiological questionnaires accurately. Further studies were required to determine the weight of lifestyle influences on the association between uric acid and renal function progression.

In conclusion, metabolic syndrome demonstrated a solid correlation with the progression of renal function, particularly in those with newly-onset MetS status. In addition to the diagnostic components of MetS, hyperuricemia could be used as a marker to identify the high risk of accelerating eGFR decline early. Furthermore, our study reiterated the significance of urate controlling for specific subgroups beyond gout and kidney stones. We suggested a potential renal benefit for the newly-onset MetS population when maintaining their serum uric acid level below the criteria for asymptomatic hyperuricemia. Clinical trials were warranted to determine whether urate-lowering treatment could halt the renal function decline of this population before it became clinically significant.

## Data availability statement

The original contributions presented in the study are included in the article/**Supplementary Material**. Further inquiries can be directed to the corresponding authors.

## Ethics statement

The studies involving humans were approved by Ethics Review Committee of Peking Union Medical College Hospital & Chinese Academy of Medical Sciences. The studies were conducted in accordance with the local legislation and institutional requirements. The participants provided their written informed consent to participate in this study.

## Author contributions

QX: Conceptualization, Data curation, Formal analysis, Investigation, Methodology, Software, Visualization, Writing – original draft. XF: Data curation, Investigation, Validation, Writing – review & editing. GC: Conceptualization, Data curation, Formal analysis, Funding acquisition, Investigation, Methodology, Software, Supervision, Validation, Visualization, Writing – original draft. JM: Data curation, Investigation, Validation, Writing – review & editing. WY: Data curation, Investigation, Validation, Writing – review & editing. SA: Data curation, Investigation, Validation, Writing – review & editing. LW: Investigation, Methodology, Supervision, Validation, Writing – review & editing. KZ: Data curation, Funding acquisition, Investigation, Supervision, Validation, Writing – review & editing. YQ: Supervision, Validation, Writing – review & editing. LC: Supervision, Validation, Writing – review & editing. ML: Project administration, Supervision, Writing – review & editing. XL: Funding acquisition, Project administration, Supervision, Validation, Writing – review & editing.
