# Supplementary material for: New-onset metabolic syndrome is associated with accelerated renal function decline partially through elevated uric acid: an epidemiological cohort study
Source: Front Endocrinol (Lausanne). 2024 Feb 2;15:1328404. doi: 10.3389/fendo.2024.1328404 (PMC10869501; doi:10.3389/fendo.2024.1328404)

## **Supplementary Table 1:** Univariate analysis of delta eGFR in follow-up population of 2008 and 2014 epidemiology studies

| **Variate** | **Statistics** | **Delta eGFR** | **P** |
| --- | --- | --- | --- |
| Metabolic syndrome status |  |  |  |
| Never | 2545 (48.84%) | 0 |  |
| Previously abnormal | 491 (9.42%) | -1.07 (-1.81, -0.33) | 0.0048 |
| New-onset | 821 (15.76%) | 1.30 (0.70, 1.90) | <0.0001 |
| Consistent | 1354 (25.98%) | -0.05 (-0.55, 0.46) | 0.8524 |
| Gender |  |  |  |
| Female | 2736 (52.36%) | 0 |  |
| Male | 2489 (47.64%) | -0.65 (-1.07, -0.24) | 0.0023 |
| Age | 55.41 ± 9.85 | 0.04 (0.02, 0.06) | 0.0007 |
| Sports doer |  |  |  |
| No | 3691 (70.64%) | 0 |  |
| Yes | 1534 (29.36%) | 0.11 (-0.35, 0.57) | 0.6356 |
| Sleep hours in 2008 | 8.64 ± 1.35 | 0.11 (-0.04, 0.27) | 0.1509 |
| Sleep hours in 2014 | 7.31 ± 1.57 | 0.14 (0.01, 0.28) | 0.0408 |
| Active drinker in 2008 |  |  |  |
| No | 3150 (60.54%) | 0 |  |
| Yes | 2053 (39.46%) | -0.61 (-1.04, -0.18) | 0.0053 |
| Active drinker 2014 |  |  |  |
| No | 4017 (77.00%) | 0 |  |
| Yes | 1200 (23.00%) | -1.23 (-1.72, -0.73) | <0.0001 |
| Active smoker 2008 |  |  |  |
| No | 3406 (65.19%) | 0 |  |
| Yes | 1819 (34.81%) | -0.63 (-1.07, -0.19) | 0.0051 |
| Active smoker 2014 |  |  |  |
| No | 4554 (87.29%) | 0 |  |
| Yes | 663 (12.71%) | -0.49 (-1.12, 0.14) | 0.1289 |
| Passive smoker 2008 |  |  |  |
| No | 3153 (60.34%) | 0 |  |
| Yes | 2072 (39.66%) | -0.28 (-0.70, 0.15) | 0.2071 |
| Passive smoker 2014 |  |  |  |
| No | 2850 (54.63%) | 0 |  |
| Yes | 2367 (45.37%) | -0.69 (-1.11, -0.27) | 0.0014 |
| Renal diseases 2008 |  |  |  |
| No | 4134 (79.17%) | 0 |  |
| Yes | 1088 (20.83%) | -0.40 (-0.91, 0.12) | 0.1308 |
| Renal diseases 2014 |  |  |  |
| No | 4454 (85.37%) | 0 |  |
| Yes | 763 (14.63%) | -0.05 (-0.64, 0.54) | 0.8715 |
| Kidney stone 2008 |  |  |  |
| No | 4830 (92.44%) | 0 |  |
| Yes | 395 (7.56%) | -1.01 (-1.80, -0.22) | 0.0123 |
| Kidney stone 2014 |  |  |  |
| No | 4734 (90.74%) | 0 |  |
| Yes | 483 (9.26%) | -0.88 (-1.60, -0.16) | 0.0173 |
| Cardiovascular diseases 2008 |  |  |  |
| No | 4834 (92.52%) | 0 |  |
| Yes | 391 (7.48%) | -0.02 (-0.81, 0.78) | 0.9646 |
| Cardiovascular disease 2014 |  |  |  |
| No | 4754 (91.14%) | 0 |  |
| Yes | 462 (8.86%) | 0.37 (-0.37, 1.11) | 0.3244 |
| Gout 2008 |  |  |  |
| No | 5199 (99.75%) | 0 |  |
| Yes | 13 (0.25%) | -1.77 (-5.96, 2.42) | 0.4084 |
| Urinary tract infection 2008 |  |  |  |
| No | 4524 (86.63%) | 0 |  |
| Yes | 698 (13.37%) | -0.04 (-0.66, 0.57) | 0.8932 |
| Urinary tract infection 2014 |  |  |  |
| No | 4964 (95.15%) | 0 |  |
| Yes | 253 (4.85%) | 0.35 (-0.62, 1.33) | 0.4789 |
| Cerebrovascular diseases 2008 |  |  |  |
| No | 4986 (95.43%) | 0 |  |
| Yes | 239 (4.57%) | 0.83 (-0.18, 1.83) | 0.1062 |
| Cerebrovascular disease 2014 |  |  |  |
| No | 4834 (92.68%) | 0 |  |
| Yes | 382 (7.32%) | 1.53 (0.73, 2.33) | 0.0002 |
| Family history of obese |  |  |  |
| No | 4592 (87.89%) | 0 |  |
| Yes | 633 (12.11%) | -0.63 (-1.27, 0.01) | 0.0546 |
| Family history of hypertension |  |  |  |
| No | 3059 (58.55%) | 0 |  |
| Yes | 2166 (41.45%) | -0.22 (-0.65, 0.20) | 0.3086 |
| Family history of diabetes |  |  |  |
| No | 4579 (87.64%) | 0 |  |
| Yes | 646 (12.36%) | -0.10 (-0.73, 0.54) | 0.7650 |
| Family history of hyperlipidemia |  |  |  |
| No | 5221 (99.92%) | 0 |  |
| Yes | 4 (0.08%) | 6.13 (-1.43, 13.69) | 0.1120 |
| Family history of gout |  |  |  |
| No | 5223 (99.96%) | 0 |  |
| Yes | 2 (0.04%) | -2.89 (-13.59, 7.80) | 0.5959 |
| Family history of CVD |  |  |  |
| No | 4096 (78.39%) | 0 |  |
| Yes | 1129 (21.61%) | 0.02 (-0.49, 0.53) | 0.9380 |
| Family history of CBD |  |  |  |
| No | 3331 (63.75%) | 0 |  |
| Yes | 1894 (36.25%) | -0.38 (-0.82, 0.05) | 0.0851 |
| Family history of CKD |  |  |  |
| No | 5074 (97.11%) | 0 |  |
| Yes | 151 (2.89%) | -0.13 (-1.38, 1.12) | 0.8339 |
| Family history of cancer |  |  |  |
| No | 4454 (85.24%) | 0 |  |
| Yes | 771 (14.76%) | -0.58 (-1.16, 0.01) | 0.0559 |
| BMI 2008 | 25.25 ± 3.54 | -0.09 (-0.14, -0.03) | 0.0047 |
| BMI 2014 | 26.03 ± 3.65 | -0.03 (-0.08, 0.03) | 0.3576 |
| Mean MAP in 2008 | 102.00 ± 12.30 | 0.04 (0.03, 0.06) | <0.0001 |
| Mean MAP in 2014 | 108.08 ± 13.42 | 0.00 (-0.01, 0.02) | 0.5335 |
| Albumin (g/L) 2008 | 46.81 ± 2.60 | -0.45 (-0.53, -0.37) | <0.0001 |
| Albumin (g/L) 2014 | 44.61 ± 2.44 | -0.25 (-0.34, -0.17) | <0.0001 |
| Serum creatinine 2008 | 63.88 ± 13.98 | -0.15 (-0.17, -0.14) | <0.0001 |
| Serum creatinine 2014 | 66.49 ± 29.56 | 0.07 (0.07, 0.08) | <0.0001 |
| Uric acid 2008 | 247.47 ± 70.83 | -0.01 (-0.02, -0.01) | <0.0001 |
| Uric acid 2014 | 272.06 ± 78.53 | 0.01 (0.01, 0.02) | <0.0001 |
| Glucose 2008 | 5.70 ± 1.52 | -0.16 (-0.29, -0.02) | 0.0257 |
| Glucose 2014 | 6.29 ± 1.80 | -0.19 (-0.31, -0.08) | 0.0011 |
| Total cholesterol 2008 | 5.01 ± 0.97 | -0.50 (-0.72, -0.29) | <0.0001 |
| Total cholesterol 2014 | 4.85 ± 0.91 | -0.02 (-0.25, 0.21) | 0.8767 |
| Triglyceride 2008 | 1.52 ± 1.45 | -0.22 (-0.36, -0.07) | 0.0030 |
| Triglyceride 2014 | 1.41 ± 1.29 | -0.11 (-0.27, 0.05) | 0.1763 |
| HDL 2008 | 1.34 ± 0.33 | 0.34 (-0.30, 0.99) | 0.2971 |
| HDL 2014 | 1.34 ± 0.32 | -0.53 (-1.18, 0.11) | 0.1066 |
| LDL 2008 | 3.08 ± 0.82 | -0.48 (-0.73, -0.22) | 0.0002 |
| LDL 2014 | 3.00 ± 0.78 | 0.03 (-0.24, 0.29) | 0.8528 |
| New-onset CKD in 2008 |  |  |  |
| No | 4705 (90.05%) | 0 |  |
| Yes | 520 (9.95%) | 1.83 (1.13, 2.53) | <0.0001 |
| New-onset CKD in 2014 |  |  |  |
| No | 4661 (89.19%) | 0 |  |
| Yes | 564 (10.81%) | 1.76 (1.08, 2.43) | <0.0001 |
| Urine ACR (mg/g) 2008 | 17.20 ± 48.90 | 0.01 (0.01, 0.02) | <0.0001 |
| Urine ACR (mg/g) 2014 | 26.74 ± 160.10 | 0.01 (0.01, 0.01) | <0.0001 |
| Urine ACR fluctuation | 9.62 ± 151.83 | 0.01 (0.01, 0.01) | <0.0001 |

**Supplementary Figure 1**: The distribution of delta eGFR in each MetS status subgroup.


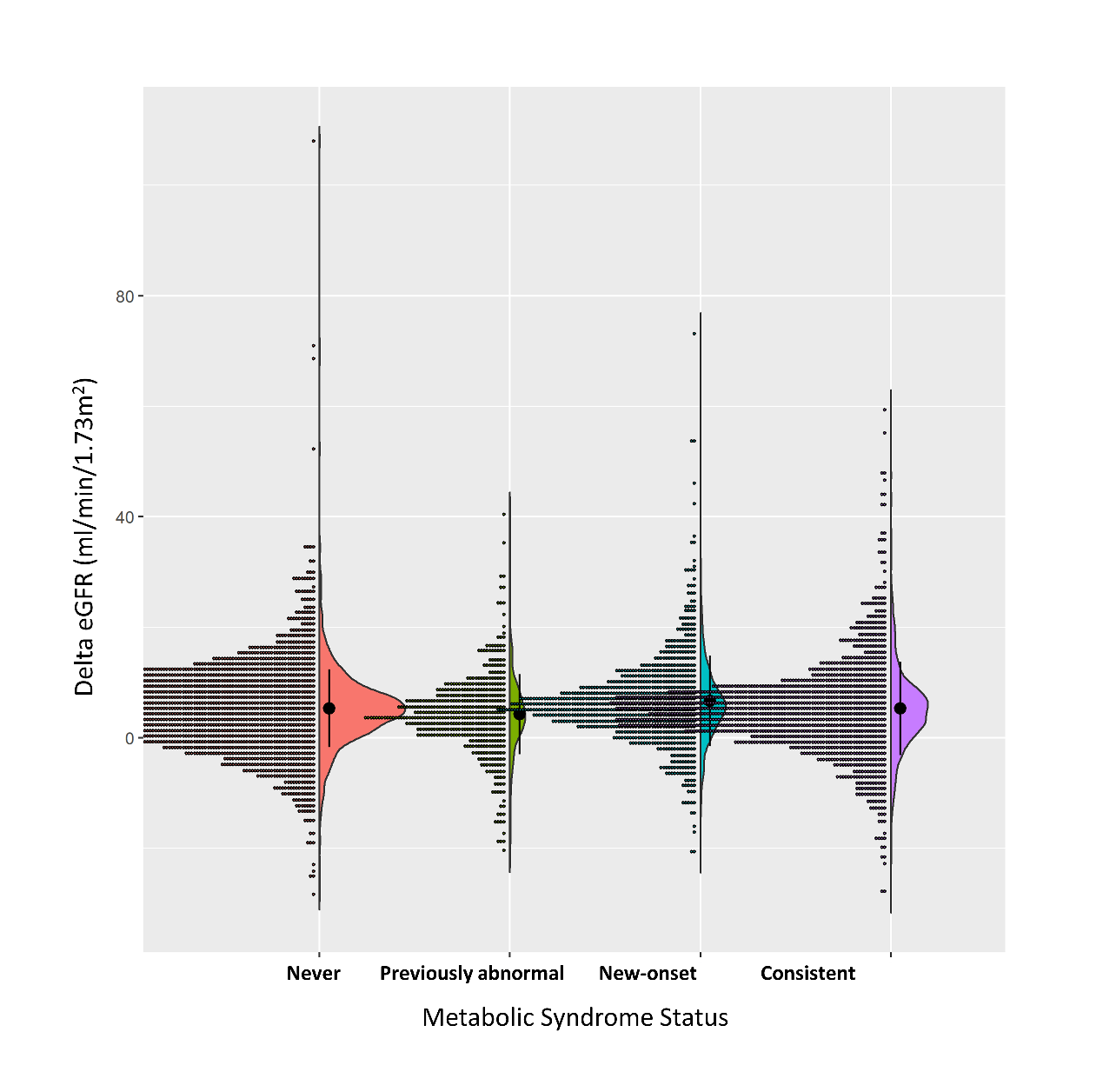


**Supplementary Figure 2**: The association between uric acid in 2014 and delta eGFR.

The solid red line represents the smooth curve fit between variables. Blue bands represent the 95% confidence interval from the fit. Model adjusted for: Gender; Age at 2014; BMI 2008; Mean MAP 2008; Urine ACR (mg/g) enzyme 2008; Albumin (g/L) 2008; SCr enzyme 2008; Glucose 2008; LDL 2008; TG 2008; Uric acid 2008; Active drinker; Kidney stone 2008; Albumin 2014; Glucose 2014; Urine ACR (mg/g) 2014; Active drinker 2014; Kidney stone 2014; Passive smoker 2014; Cerebrovascular disease 2014; Smoker 2008 recoded; Sleep hours 2014; Urine ACR fluctuation


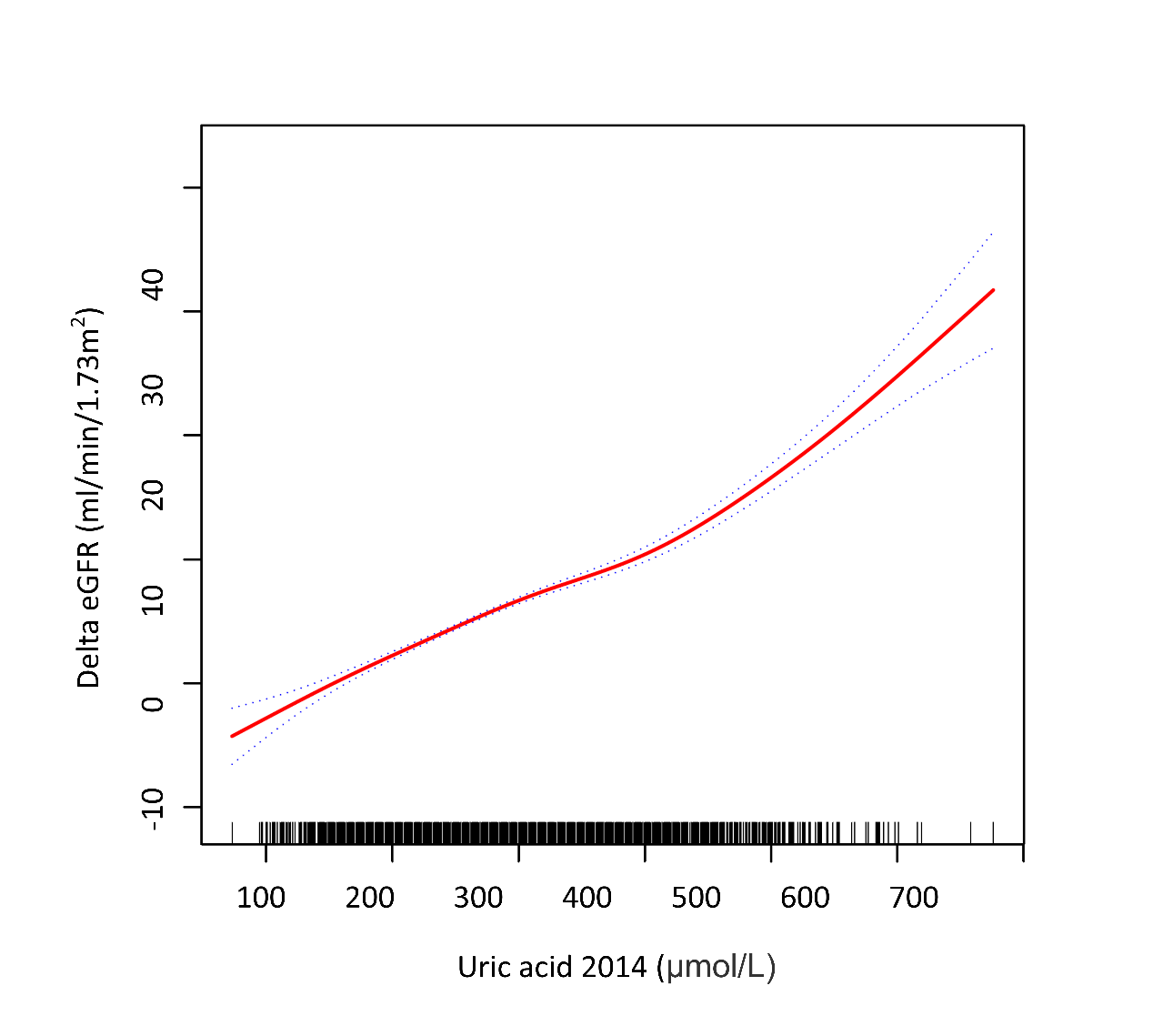


**Supplementary Figure 3**: The association between uric acid in 2014 and delta eGFR in different metabolic syndrome status subgroups.


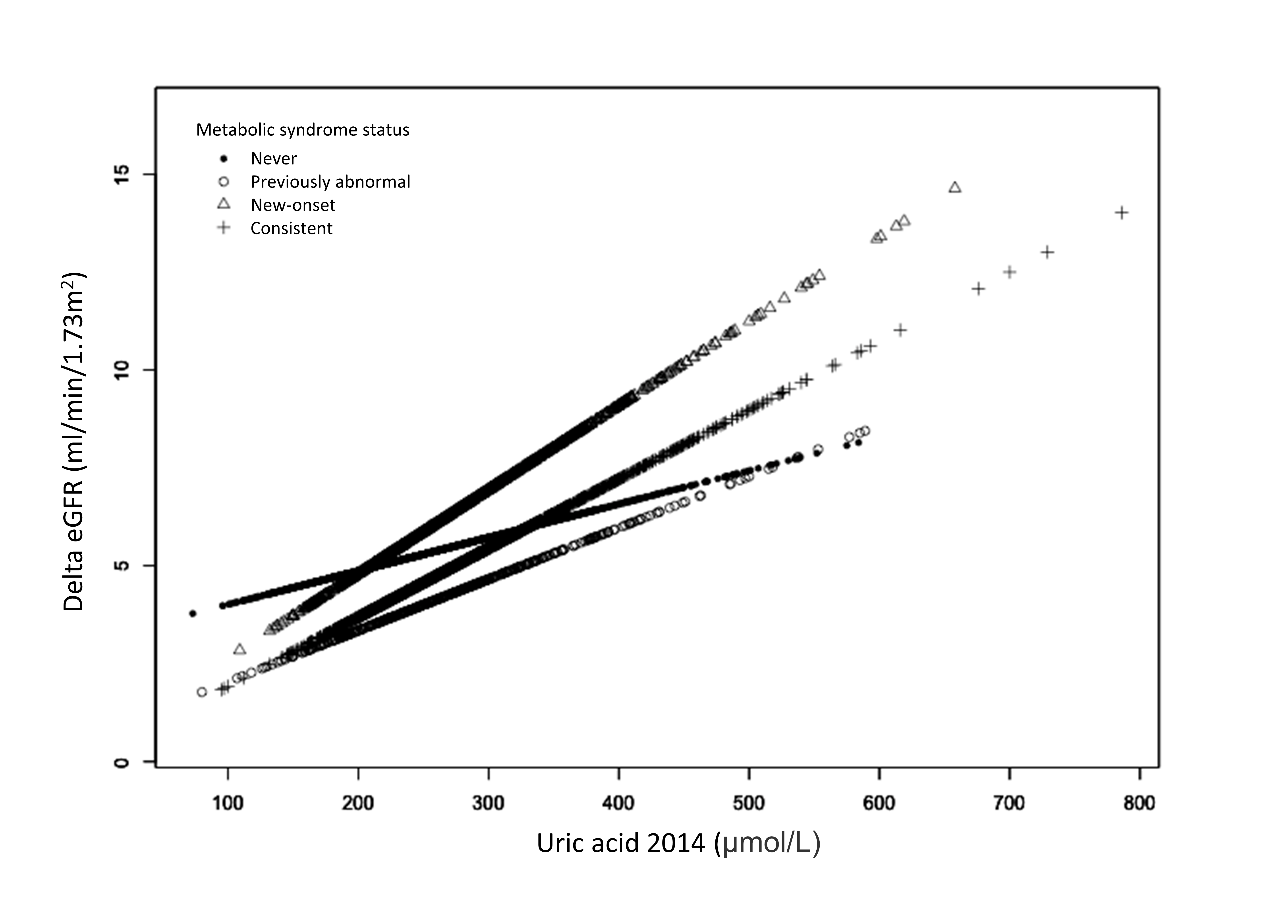

Supplement: Supplementary file 1 [file DataSheet_1.docx]
